# Atomic Resolution Electron Microscopy: A Key Tool for Understanding the Activity of Nano-Oxides for Biomedical Applications

**DOI:** 10.3390/nano11082073

**Published:** 2021-08-16

**Authors:** Alberto Azor-Lafarga, Isabel Gómez-Recio, M. Luisa Ruiz-González, José M. González-Calbet

**Affiliations:** 1 Departamento de Química Inorgánica, Facultad de Químicas, Universidad Complutense, CEI Moncloa, 28040 Madrid, Spain; aazorlaf@ucm.es (A.A.-L.); isabelgomezrecio@ucm.es (I.G.-R.); luisarg@ucm.es (M.L.R.-G.); 2 ICTS ELECMI Centro Nacional de Microscopia Electrónica, Universidad Complutense, 28040 Madrid, Spain

**Keywords:** transition metal oxides, electron microscopy, atomic resolution, EELS, hollandite related mangnese oxides

## Abstract

Transition metal oxides constitute one of the most fruitful sources of materials with continuously increasing potential applications prompted by the expectations derived from the reduction of the particle size. The recent advances in transmission electron microscopy, because of the development of lenses, have made it possible to reach atomic resolution, which can provide answers regarding the performance of the transition metal nano-oxides. This critical information is related not only to the ability to study their microstructural characteristics but also their local composition and the oxidation state of the transition metal. Exploring these features is a well-known task in nano-oxides for energy and electronic technologies, but they are not so commonly used for elucidating the activity of these oxides for biomedical applications. Nevertheless, the identification at the atomic level of a certain dopant or the unambiguous determination of the oxidation state of a transition metal in a nano-oxide can be important questions to be answered in a certain biomedical application. In this work, we provide several examples in transition metal nano-oxides to show how atomic-resolution electron microscopy can be a key tool for its understanding.

## 1. Introduction

Transition metal oxide (TMO) nanomaterials have attracted great interest in comparison with other counterparts due to their remarkable and fruitful physical [1], chemical [2], and mechanical properties [3]. These properties arise from their unique structural and redox features that can be tuned to provide improved performances. Therefore, TMOs can potentially be used in a wide range of different applications in areas of crucial interest for the society such as energy technologies, electronic devices, and biomedicine. Among them, the use of TMOs for biomedical purposes involves challenging areas such as implants, drug-delivery systems, cancer diagnosis and therapy, antimicrobials, and contrast agents. Such fascinating applications require multidisciplinary efforts from basic and applied sciences. At the root of these developments are the synthesis and characterization of TMOs. Preparing TMO materials with the adequate morphology, size, degree of agglomeration, functionalization, metal transition oxidation states, among others, are critical factors. Transmission electron microscopy (TEM) can play an important role in unveiling details of these TMOs going from their textural properties to the atomic resolution.

### 1.1. Titanium Oxide

**Figure 1:**
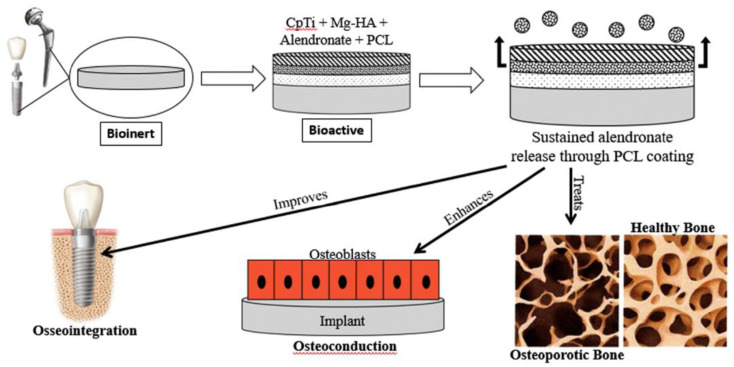
Hydroxyapatite (HA)-coated commercially pure titanium (CpTi) is initially bioinert, but the deposition of a Mg–HA coating and loaded with alendronate enhances bioactivity. Polycaprolactone (PCL) coating enables the sustained release of alendronate, which can be used in hips and dental implants to treat and prevent osteoporosis, enhance osteoconduction and improve osseointegration. Images: Wolgin, MD, orthopedic surgeon. The Osteoporosis Center. American Academy of Implant Dentistry. Adapted with permission from [10]. Copyright Elsevier.

Titanium oxide might be the most common TMO from those that can be directly implemented for many healthcare applications. Titanium is widely used as a material for permanent implants in orthopedic and dental applications. It is well established that Ti shows adequate osseointegration, i.e., mechanically stable interface towards bone. The good biomimetic properties are due to the beneficial properties of a 2–7 nm titanium oxide layer formed onto metallic Ti when exposed to oxygen [4]. Besides its stability and lack of toxicity in the physiological environment, TiO_2_ increases calcium interactions, which are important for protein and subsequent osteoblast adhesion [5]. However, enhanced bonding can be achieved using bioactive materials that form a perdurable union with bone through the spontaneous formation of hydroxyapatite (HA) on their surface. The HA layer improves the integration of the implant in the organism acting as a bonding layer to the bone at an atomic/molecule level. For this reason, HA coatings of implants are proposed for the stronger early fixation of the uncemented prostheses. Although HA coatings are chemically stable and show long-term survival, there are several concerns about their load bearing performance [6]. Possible ways to solve this lack of mechanical stability could be inter-growing the HA with TMOs such as zirconia [7] and alumina [8] or be constituting composites with graphene [9]. Moreover, doping HA with other bioactive ions, such as Ag^+^ [6] and Mg^2+^ [10], could introduce interesting bactericide and other therapeutic effects (Figure 1).

The correct performance of these devices depends, as previously mentioned, on their proper attachment at an atomic scale. Here is where the atomic-resolution TEM becomes an indispensable tool to understand which issues the material should address before it can be put into use. Here, the main efforts must be focused on the understanding of the biomaterial surface and how it behaves in contact with tissues. Since the natural response of the body to a foreign material is to encapsulate it with a fibrous material, interactions between the body and the biomaterial are mediated by this fibrous layer. Initial interactions occur between the biomaterial surface, water, ionic species, and organic molecules, which then mediate further interactions with body tissues. Surface engineering can influence these interactions and hence, improve the biocompatibility of the biomaterial. Both at in vivo and in vitro conditions, whenever titanium oxide is in contact with an aqueous environment, it will lead to the hydroxylation of the surface via the dissociative adsorption of water molecules [11,12]. Computational studies show that the hydroxylation degree of TiO_2_ is conditioned by its structure, whether it is rutile, anatase, or amorphous, and surface structural relaxations [13,14]. The amount of hydroxyl groups and their nature modify the surface charge and, subsequently, the hydrophobicity of the material. As well as water molecules, the superficial hydroxyl groups also interact with the ions solvated in the water. Again, ab initio studies performed for ions present in blood plasma evidence the importance of the deep knowledge and the control of the biomaterial structure and surface to understand how they interact with them [15,16]. Moreover, further interactions with biomolecules seem to be governed by the first hydrated layer [5].

All these crucial effects have been predicted by computational methods. Among the techniques that can help us have an empirical evidence of them, atomically resolved TEM is the only one that provides a direct observation. While scanning electron microscopy (SEM) images are widely used in the literature for a direct macroscopic visualization of bone–implant interfaces [6,16,17], TEM is scarcely found [15] and scanning transmission electron microscopy (STEM) and associated spectroscopic analysis are even rarer [9].

### 1.2. Magnetic Nanoparticles (MNPs)

TMOs constitute a widely studied family of functional materials due to the varied compositions and properties that they exhibit. Among them, magnetism is an outstanding one. Over the past decades, the biomedical use of MNPs has become very popular as evidenced by a large number of publications introducing novel synthesis techniques, discussing new application strategies and reporting successful in vitro and in vivo application results [18]. Among these applications, magnetic resonance imaging (MRI), targeted drug delivery, and magnetic hyperthermia are the most promising ones.

In MRI, which is a non-invasive diagnosis technique, MNPs are used as contrast enhancement agents [18]. Paramagnetic contrast agents have been used for a long time, but recently, superparamagnetic iron oxide nanoparticles (SPIOs) have been found to influence MRI contrast as well. Unlike paramagnetic contrast agents, SPIOs can be functionalized and size-tailored in order to adapt to various kinds of soft tissues. Although both types of contrast agents have an inducible magnetization, their influence mechanisms on spin–spin and spin–lattice relaxation of protons are different [19,20].

Nanoparticles (NPs) designed for drug delivery should be, above all, biodegradable and biocompatible; SPIOs fulfil these two requirements, and that is why they have garnered special interest [21]. Due to their unique physical properties and ability to function at the cellular and molecular levels of biological interactions, MNPs have been actively investigated as the next generation of targeted drug delivery for more than 30 years [22]. The aim of targeted drug delivery and therapy is to transport a drug directly to the area of the disease under various conditions and thereby treat it deliberately, with lower side effects [23]. The greatest therapeutic potential is probably associated with applications involving the so-called smart particles, constituted by a magnetic core (to direct particles to the vicinity of the target and also for hyperthermia or for drug temperature-enhanced release), a recognition layer (where suitable receptors are attached), and a therapeutic load (adsorbed inside the pores or hosted within the internal cavities of the particles) [24]. Iron oxides, mainly Fe_3_O_4_ (magnetite) and γ-Fe_2_O_3_ (maghemite), with a core–shell structure, are the most widely used magnetic materials [25,26]. Nevertheless, other iron-based compounds can be found in the literature. Carbonyl iron, which is a well-known material with a unique form of elemental iron thanks to its small particle size, is also used as a magnetic core [27]. β-FeOOH (akaganeite), an iron oxyhydroxide with a tunnel hollandite structure, has been also tried as a drug delivery vector [28] and as MRI contrast agent [29,30]. The characteristics of this system will be discussed further in this work.

MNPs designed for hyperthermia treatments face the same issues described above. In this procedure, suitable MNPs are placed under an alternating-current (AC) magnetic field and due to magnetic friction, and the heat released from them results in the reduction of the cancerous tissue. The optimum temperature range for this process is 41–46 °C [31]. However, even though the efforts made in the recent years have been intensively focused on developing a high potential MNP agent for in vivo hyperthermia, there has been no successful report that observed clinically suitable self-heating temperature-rising characteristics [32]. To improve this performance, different synthesis methods have been tested [33] in combination with different degrees of substitution of iron by other magnetically active transition metal elements such as Co [34,35], Ni [36], and Mn [37]. A comparative study, using samples with similar particle size and coordinated molecules on the NPs surface, seems to evidence that Mn doped phases show the better performance, but biocompatibility and cytotoxicity studies are still required [38]. Atomically resolved TEM and associated spectroscopies constitute an ideal tool for identifying these compositional changes and to understand their role in hyperthermia process.

**Figure 2:**
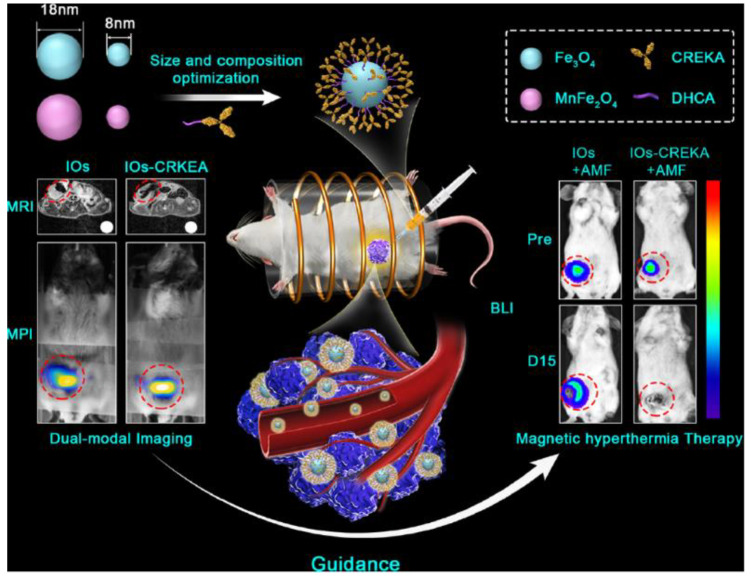
Schematic illustration of the developed CREKA-modified iron oxide (IO) nanoparticles (NPs) with different sizes and compositions for the potential application on dual-mode RI/MPI and magnetic hyperthermia therapy for the precise cancer imaging and therapy on a 4T1 breast tumour mouse model. Adapted with permission from [39]. Copyright (2019) American Chemical Society.

In summary, the high flexibility of MNPs lies in their simultaneous ability to the following: (i) coordinating targeting molecules and antitumoral drugs; (ii) acting as MRI contrast agents, and (iii) producing a hyperthermia effect. Therefore, all these characteristics are combined in a single material based on TMOs (Figure 2). For instance, the drug delivery performance can be improved by heating the MNPs thanks to magnetic friction [39], while the same MNPs producing a hyperthermic response can be monitored by MRI [40].

**Figure 3:**
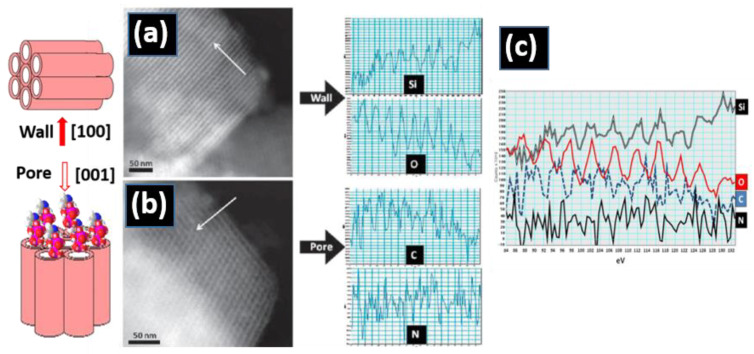
Scanning transmission electron microscopy (TEM)-high-angle annular dark-field (HAADF) images of an SBA-15 mesoporous matrix loaded with zoledronate molecules along the wall, the [100] direction, the pore, and the [001] direction and the corresponding electron energy loss spectroscopy (EELS) intensity profiles (see arrows): (**a**) along the [100] direction, the EELS profile shows the presence of Si and O; (**b**) along the [001] direction, the pores were observed, and the profile revealed the presence of N and C inside; (**c**) the Si, O, C, and N profiles along the same direction, the [001] direction [49].

The morphological and basic microstructural characterization of these systems has been addressed by low- and medium-resolution TEM [41,42,43,44,45] to obtain information on the particle size and degree of crystallinity. Further and more precise structural and compositional information can be achieved by high-resolution transmission electron microscopy (HRTEM) using state-of-the-art instruments. During the last two decades, HRTEM has experienced a real breakthrough due to the development of aberration correctors [46,47,48], which allow the acquisition of atomically resolved images, using either a parallel beam illumination or a convergent one (STEM). The manufacturing of small probes is also very important for the chemical analysis at atomic resolution in combination with electron energy loss spectroscopy (EELS) and energy dispersive X-ray spectroscopy (EDS). For instance, the confinement of drug molecules in mesoporous matrices has been confirmed using a combination of STEM and EELS techniques [49]. In fact, EELS experiments proved the presence of zoledronate molecules inside an SBA-15 mesoporous matrix. This can be observed in Figure 3, where STEM-HAADF images of this system along [100] and [001] directions are displayed with the corresponding EELS intensity profiles, evidencing the presence of Si and O across the wall (see Figure 3a) and C and N, characteristic of the zoledronate molecules, along the pore direction (see Figure 3b). Along this profile, Si and O signal appeared at the same position (white contrast) alternating with C and N (dark contrast), proving that zoledronate was inside the pore, but not in the mesoporous wall (see Figure 3c).

### 1.3. Other Applications of Transition-Metal-Based NPs

Several transition metals such as Fe [50], Cu [51], Zn [52], Mo [53], and V [54], whether as free-standing ions or as small metallic clusters dispersed in, for example cytoplasm or blood, are essential mineral nutrients for a correct human biological process [55]. These elements are naturally present in many products consumed by humans but can be artificially incorporated as TMOs. The therapeutic effects of these elements can also improve or complement the performance of other biomaterials when incorporated in their structures [56,57,58,59].

### 1.4. Manganese Oxides

We have left manganese functional oxides for the end, because we will discuss in depth some of their phases further in this work. Manganese is a very important cofactor in several key enzymes for a correct brain function, such as manganese superoxide dismutase and glutamine synthetase [60]. Besides, the supplementation of manganese increases the serum osteocalcin and bone mass density in ovariectomized mice, suggesting manganese has an impact in bone hemostasis [61]. Interestingly, manganese ion has been shown to increase osteoblast proliferation [62]. Additionally, doping alumina implants with manganese enhances the proliferation of bone marrow compared to undoped controls. The porous nature of the scaffold allows for tissue ingrowth, suggesting that this mineral is a valuable addition to bone–ceramic interfaces [63]. However, manganese has plenty of oxidation states, so it would be interesting to understand the effect of charges on tissue regeneration. Moreover, the exact mechanism by which manganese improves bone mineralization is not well understood. Thus, additional studies on this element are required and, again, atomically resolved TEM in combination with local spectroscopic techniques could provide the answer for these questions.

Manganese oxide NPs have been utilized as contrast agents [64] and drug delivery vehicles [65]. More recently, the anti-tumoral activity of MnO_2_ NPs has been described, triggering intense research interest [66]. In solid tumors, hypoxia (low oxygenation) often occurs because of a disrupted balance between the supply and consumption of O_2_, owing partially to tumor growth and vascular abnormalities, the latter also affecting O_2_ transport insufficiencies. This causes, on the one hand, the switch from aerobic to anaerobic metabolism in hypoxic tumors for energy preservation by activating glucose transporters and glycolytic enzymes, leading to an increase in levels of lactic acid and acidosis. On the other hand, it also produces excess amounts of reactive oxygen species (ROS), such as hydrogen peroxide (H_2_O_2_). The combined effect of hypoxia, acidosis, and ROS promote mutagenesis, metastasis of cancer cells, angiogenesis, and resistance to therapies, contributing to treatment failure. MnO_2_ NPs catalyze H_2_O_2_ decomposition in a highly efficient and specific way. This has two simultaneous therapeutic effects. On one side, reduces hypoxia through O_2_ production into the tumor during a prolonged time. On the other side, reduces ROS concentration is reduced, and pH is regulated [67]. In the latter case, the MnO_2_ NPs are decomposed to harmless, water-soluble Mn^2+^ ions, avoiding the in vivo accumulation of the metal oxide commonly observed for other metal-based NP systems.

MnO_2_ therapeutical effects can be combined with its imaging abilities [46,68]; however, the most promising and effective strategy seems to be the association of its anticancer drug delivery properties in conjunction with its tumoral environment regulation, both of which are intrinsic features of this oxide [69,70]. Recently, some other interesting properties of the MnO_2_ have been reported. Hence, it seems to be a promising agent in cancer phototherapy [71], where MnO_2_ acts as a photosensitizer. Some allotropes can also be used in fluorescence imaging. Here, fluorescence is quenched by MnO_2_ before entering the target cells. After entering the recipient cells, the binding of the aptamer to its target attenuates the adsorption of the aptamer on the nanosheet, and part of the fluorescence is recovered [72].

Most of MnO_2_ potential beneficial effects for biomedical purposes, such as biosensing, drug delivery and photothermal therapy, relay on its oxidizing character that enables the reduction process to Mn^2+^. Having analytical tools for testing the oxidation state of Mn is, therefore, highly desirable. EELS is an excellent technique to gather this information, especially if a highly focused beam is available, in aberration-corrected STEM microscopes, in combination with a good energy resolution, at 0.1–0.3 eV, which allow detecting small energy loss differences among the different Mn oxidation states.

In conclusion, TMOs have been extensively investigated and used for biomedical purposes thanks to their wide range of properties. Nevertheless, as pointed out in the introduction, several questions need to be addressed:

(i) Direct visualization of implant/tissue interfaces with atomic resolution in order to understand how to optimize the interactions taking place;
(ii) A compositional analysis at an atomic level of biomaterials. The local distribution of some crucial bioactive dopants included in the materials would help to enlighten their functionalities.
(iii) The behavior of biomedical TMOs is highly influenced by the oxidation state of the transition metal. Local analysis, mainly on the surface of the material, would allow deeper knowledge of how they will work.

The aim of the following sections is to show how TEM can provide structural and compositional information in some TMOs such as those presented in the introduction section above. Ranging from low-magnification imaging in conventional TEM up to atomically resolved images in aberration-corrected microscopes, important morphological and microstructural features can be unveiled in combination with the study of their composition and transition metal oxidation states through associated EDS and EELS.

## 2. The Role of the Electron Microscopy

As previously mentioned, the control of the structural and compositional details of a biomaterial can be a critical factor for the development of a certain functionality. TEM and associated spectroscopic techniques are fundamental tools to study the homogeneity, morphology, size, degree of agglomeration, composition, and functionalization of TMO NPs. This section deals with several examples of characterization of nano-oxides, starting from basic TEM and going towards atomic resolution. In conventional TEM and HRTEM, a parallel beam illuminates the sample, and the image is formed, thanks to the objective lens placed after the sample position, leading to important textural and structural information depending on the resolution. In the STEM mode, a convergent beam is focused in a small probe on the sample and scanned over the region of interest. At each scanning position, the beam is transmitted, and the scattered electrons are recorded using different detectors. Besides the structural information, very useful compositional information is obtained using a high-angle annular dark-field (HAADF) detector that is usually complemented with EELS. The resolutions of both parallel and convergent beams depend on several factors, but the aberration of the lenses is a critical one that greatly limits the attainable resolution. This problem could not be overcome, until the development of aberration correctors [47,48] enable the access to the atomic resolution.

### 2.1. Basic Microstructural Characterization of TMOs by TEM

**Figure 4:**
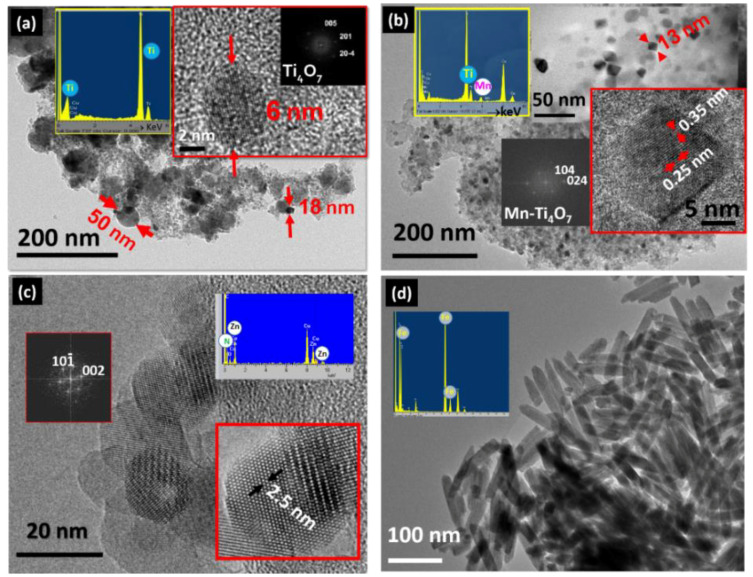
Characteristic low- and medium-magnification TEM images together with the typical EDS spectra of TiO_2_ reduced NPs (**a**); (**b**) Mn-doped TiO_2_-reduced NPs; (**c**) ZnO-functionalized NPs; (**d**) β-FeOOH nanowires.

Low- and medium-resolution TEM was used to study the homogeneity, morphology, and size. In parallel, the average composition of the NPs was measured by EDS, using the X-Ray generated when the electron beam striked the sample. Figure 4 shows TEM images and EDS information for several systems of nano-TMO mentioned in the introduction section that can illustrate this. Figure 4a,b shows an example of reduced TiO_2_ NPs, undoped and Mn-doped, prepared by a combination of sol-gel and carboreduction procedures [73,74]. The TEM study revealed that the particle size decreased when Mn was introduced (confirmed by EDS analysis) and, at the same time, a more faceted morphology was obtained. A characteristic low-magnification TEM and HRTEM image of ZnO NPs prepared by sol−gel and subsequently capped with dodecanothiol molecules is shown in Figure 4c [75]. They exhibited an average size around 20 nm as well as a spherical morphology. The presence of N in the EDS spectra signal could be related to the amine. Typical β-FeOOH nanowires obtained from the hydrolysis of FeCl_3_∙6H_2_O are depicted in Figure 4d. Homogeneously elongated particles with a width of 20 nm and a length of around 100 nm are shown. The EDS analysis confirmed the Fe presence and some remaining chloride from the precursors.

**Figure 5:**
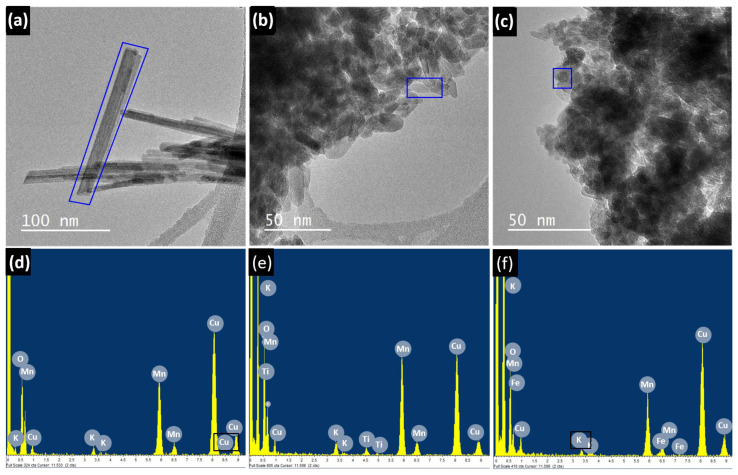
Low-magnification TEM images corresponding to the undoped sample (**a**); (**b**) the sample doped with 5% Ti; (**c**) the sample doped with 15% Fe; (**d**–**f**) corresponding EDS spectra.

Recently, optimized synthetic pathways have led to doped hollandite K_x_Mn_1−y_M_y_O_2_ (M = Fe, Ti, and Ce) [76]. The undoped K_x_MnO_2_ sample was made of nanowire-shaped particles with a typical width of around 10–20 nm and a length of 200 nm, as observed in Figure 5a. The dopant introduction gave rise to the reduction of the particle size. As a representative example, the characteristic low-magnification TEM images of Fe- and Ti-doped samples are included in Figure 5b,c, respectively. The EDS (Figure 5d–f) analysis evidences the presence of Ti and Fe in global areas shown in Figure 5b,c, in comparison with the undoped hollandite in Figure 5a.

**Figure 6:**
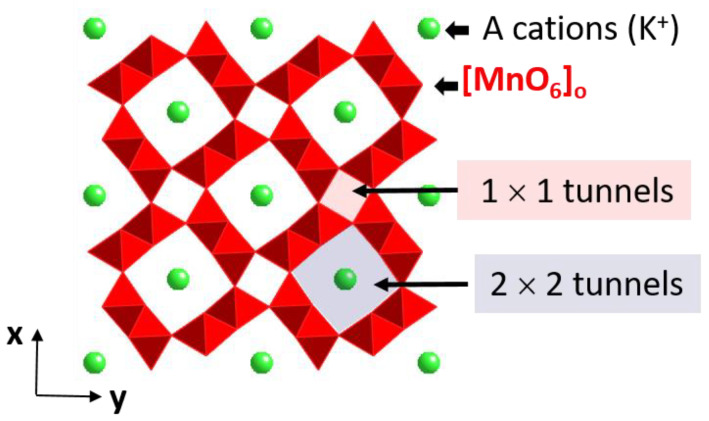
Schematic representation of the hollandite structure.

Most of the functionalities of these oxides were primarily based on their structural features. For instance, the hollandite type can be described as edge sharing [MO_6_] octahedral units which share corners to form M_8_O_16_ frameworks with two types of tunnels, i.e., 2 × 2 and 1 × 1, as shown in Figure 6.

The 2 × 2 tunnels are usually occupied by large cations such as K^+^, Pb^2+^, and Ba^2+^, while the smaller 1 × 1 tunnels are usually empty. The inclusion of these cations modifies the Mn oxidation state in the parent MnO_2_ rendering mixed Mn^3+^ and Mn^4+^, which changes the redox behavior of these oxides. This and other properties can be also tuned by modifying cationic sublattices through dopant incorporation. In addition, the reduction of the particle size can bring about new benefits for the applications of these systems. The location of the dopants, as well as the oxidation state of the metal transition, influences the behavior of hollandites. For this reason, it is essential to obtain structural and compositional information with higher resolution.

### 2.2. Atomically Resolved Electron Microscopy

#### 2.2.1. Structural Information

**Figure 7:**
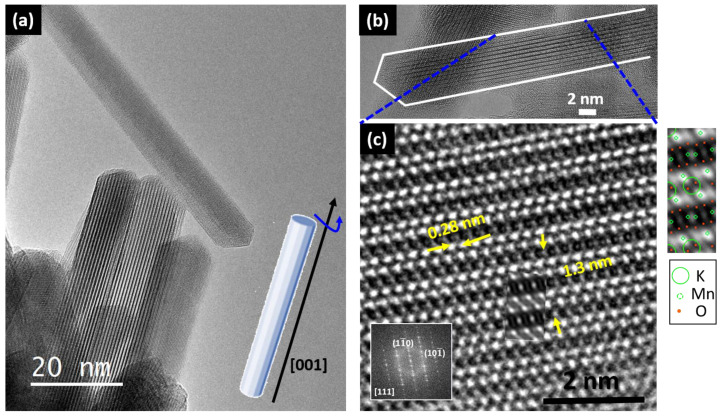
(**a**) High-resolution transmission electron microscopy (HRTEM) corresponding to several nanowires of undoped hollandite along the [11n] direction around the [001] direction as schematically represented in the model at the bottom-right; (**b**) image of one nanowire along the [111] direction; (**c**) atomically resolved detail and calculated image.

The HRTEM image of the above mentioned doped hollandites shows that elongated particles are usually oriented along [111], [112], [113] zone axes, which is related to its anisotropy (see Figure 7a). An atomic resolved TEM image of a single nanowire along [111] is shown in Figure 7b. The measured periodicities and the FFT on the enhanced detail (Figure 7c) agree with the tetragonal hollandite unit cell along this projection. Even more, the image calculation considering this cell is in good agreement with the experimental one (see inserted calculated image for a thickness Δt of 8 nm and a defocus Δf of 20 nm). The calculated image, showing the atom sites, is also depicted on the right-hand side of the experimental image.

**Figure 8:**
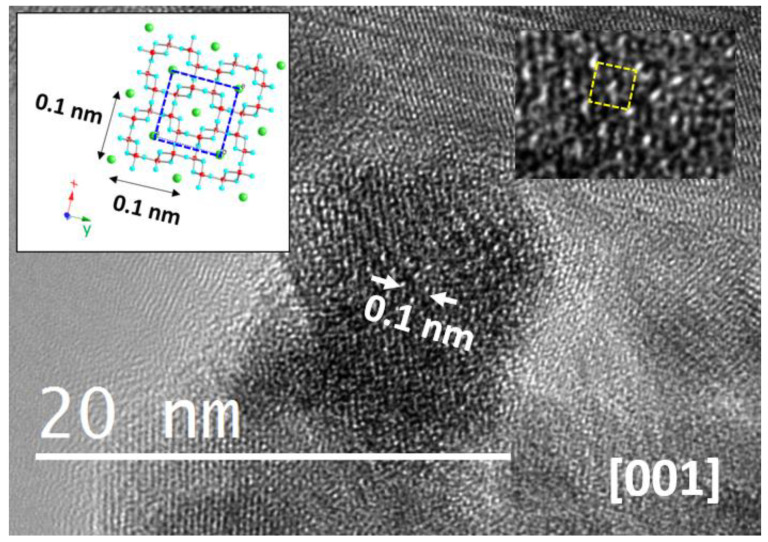
HRTEM image along the [001] direction corresponding to a 15% Fe-doped sample. An enhanced detail is shown in the right-upper part, and the schematic model along this projection is included in the left-upper part.

In order to get structural information along the [001] direction, i.e., along the nanowires perpendicular direction in the undoped counterpart, it is necessary to pack the sample in a resin and then perform cross-section cuts. However, since particle size decreases when dopants are introduced, the length-to-width ratio decreases, promoting the [001] zone axis observation. A typical image of a 15% Fe-doped hollandite is shown in Figure 8, where the schematic model of the unit cell along this direction is also included.

This structural study could be extrapolated to, for example, the TiO_2_ layer on the surface on the orthopedic implants mentioned earlier [13,14]. That would unveil the exact structure of the external surface predicted by ab initio calculations allowing improving or completing them.

#### 2.2.2. Compositional Information: STEM-HAADF-EELS Studies

##### Cation Distribution

Atomic-resolution electron microscopy can also provide answers to compositional issues. In the following, several examples of doped materials with new or improved functionalities will be discussed. The distribution of these elements along the material, even in trace concentrations, can be determined in parallel with the structural analysis. For that purpose, it is very convenient to use the STEM technique in an aberration-corrected microscope, because the electron beam is focused into a small probe, of the size of the atomic columns, which is scanned over the sample. The scattered electrons can be analyzed in several detectors that provide different kinds of information. Electrons scattered at high angles are collected in an HAADF detector. This high scattering is produced, when the electrons pass close to the atomic nuclei, and hence, the imaging contrast is highly dependent on the atomic number Z (~Z^1.8^). The atomic columns appear bright on a dark background. The higher Z is, the higher the bright contrast is, making possible the discrimination of atomic columns of heavy atoms with different atomic numbers. Simultaneous to STEM-HAADF images, it is possible to record an EELS spectrum at each position of the scanning, performing an elemental analysis as a function of the probe position. This capability allows unveiling the local composition of the doped TMO. In general, EELS measures the energy distribution of the electrons that interact with the sample and lost energy due to inelastic scattering [77]. In an EELS spectrum, three different regions can be distinguished as following: zero loss (ZL), low loss, and core loss. The ZL peak is centered at 0.0 eV, representing electrons that do not suffered inelastic scattering, without losing energy or very low losses. The low loss region arises from the inelastic scattering of the valence or conduction electrons. Energy losses above 50 eV are related to inelastic scattering from inner-shell electrons that are excited to unoccupied states above the Fermi level producing the so-called ionization edges. The onset of this edge corresponds to the ionization threshold, which is characteristic of a particular element allowing its identification.

**Figure 9:**
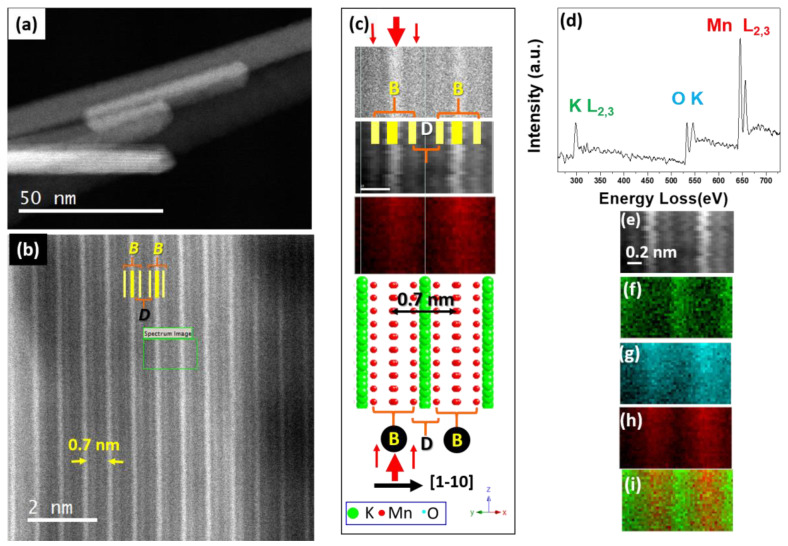
(**a**) Low-magnification STEM-HAADF image of several K_0_._12_MnO_2_ nanowires; (**b**) HAADF image detail of a single nanowire; (**c**) from top to bottom: EELS spectra; online HAADF image, Mn chemical map; schematic model along the [110] direction; (**d**)sum spectra corresponding to the area framed in green in part b; (**e**) online HAADF image; (**f**) K-L_2,3_ chemical map; (**g**) O-K chemical map; (**h**) Mn-L_2,3_; (**i**) K and Mn maps overlapped.

In our example, i.e., doped K_x_Mn_1−y_M_y_O_2_ (M: Fe and Ti), dopant cations identification was possible, because although Mn, Fe, and Ti have similar atomic numbers hindering its identification by HAADF, EELS mapping revealed its position, as well as those of K and Mn, in the NP. A low-magnification STEM-HAADF image for the undoped sample is shown in Figure 9a. The characteristic bright contrast over the dark background was observed. A higher-magnification image of a single nanowire (Figure 9b) perpendicular to the [001] zone axis revealed the presence of bright and dark fringes, labelled in the image as B and D, respectively. The bright fringes were, in fact, the combination of a brighter central fringe surrounded, at both its right and left sides, by two less bright ones. These blocks of three fringes were separated by dark fringes. The periodicity corresponding to this motif was around 0.7 nm, in agreement to the periodicity of the (110) layers (see the bottom part of Figure 9c). According to the Z contrast, B fringes must correspond to the heaviest cations, i.e., Mn (Z = 25), while the darker ones corresponded to K (Z = 19). The lower intensity at the center of the B block in comparison with the right and left surroundings can be explained by the higher Mn concentration in this central part, as can be observed in the schematic model in Figure 9c (bottom part).

Simultaneously, EELS spectra were acquired over the region framed in green in the HAADF image. The corresponding EELS sum spectra and the HAADF image of this region are depicted in Figure 9d,e, respectively. The spectra allowed identifying the characteristic K-L_23_, O-K, and Mn-L_2,3_ edges, from which the elemental maps of K (Figure 9f), O (Figure 9g), and Mn (Figure 9h) were gathered. For clarity, K and Mn maps overlapped are shown in Figure 9j. The positions of K and Mn were, then, confirmed, in agreement with the HAADF and the model in Figure 9c. For the sake of clarity, a simultaneously acquired spectra, an HAADF image, and a Mn chemical mapping are displayed also in line with the schematic representation of the structure in this projection.

**Figure 10:**
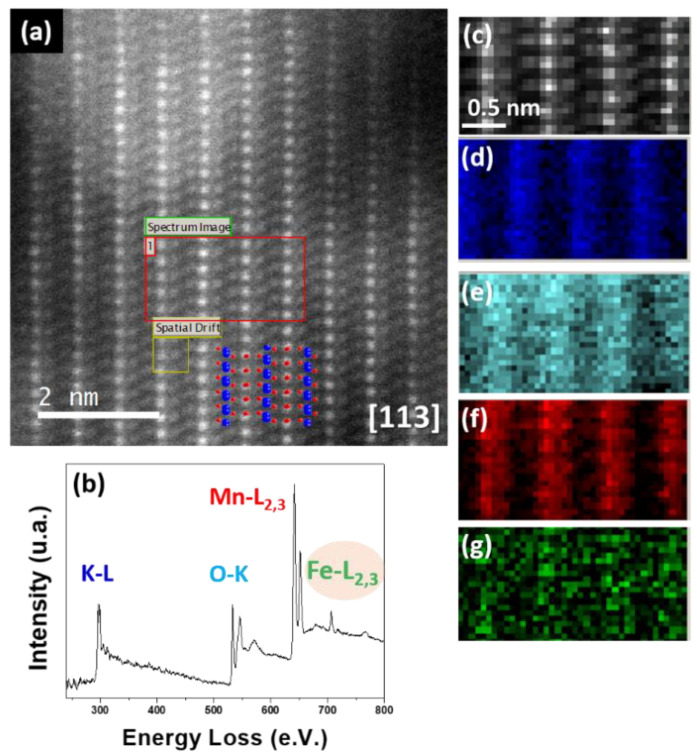
(**a**) STEM-HAADF image corresponding to K_0.09_Mn_0.88_Fe_0.12_O_2_; (**b**) sum spectra of the area framed in green in a; (**c**) simultaneously acquired HAADF image; (**d**) K-L_2,3_ chemical map; (**e**) O-K chemical map; (**f**) Mn-L_2,3_ chemical map and (**g**) Fe-L_2,3_ chemical map.

**Figure 11:**
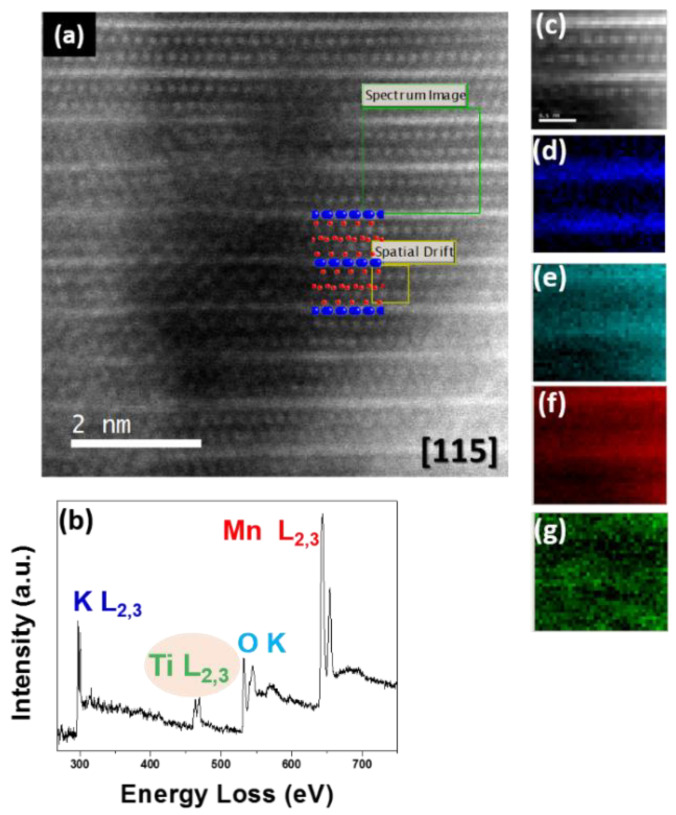
(**a**) STEM-HAADF image corresponding to K_0.11_Mn_0.92_Ti_0.08_O_2_; (**b**) sum spectra of the area framed in green in (**a**); (**c**) simultaneously acquired HAADF image; (**d**) K-L_2,3_ chemical map; (**e**) O-K chemical map; (**f**) Mn-L_2,3_ chemical map and (**g**) Ti-L_2,3_ chemical map.

Similar STEM-HAADF images were obtained for Fe- and Ti-doped samples (see Figure 10 and Figure 11). Moreover, the EELS spectra evidenced the presence of Fe (Fe-L_2,3_ around 700 eV; see Figure 10b) and Ti (Ti-L_2,3_ around 450 eV; see Figure 11b). The elemental maps indicated that Fe and Ti (Figure 10g and Figure 11g) were placed at the same atomic columns as Mn (Figure 10f and Figure 11f).

Applying a similar study to, for example, Ag- [6] or Mg [10]-doped HA or Cu-doped SiO_2_ [59], would help to elucidate how these elements work to produce their beneficial effects.

##### Oxidation States of TM by EELS

**Figure 12:**
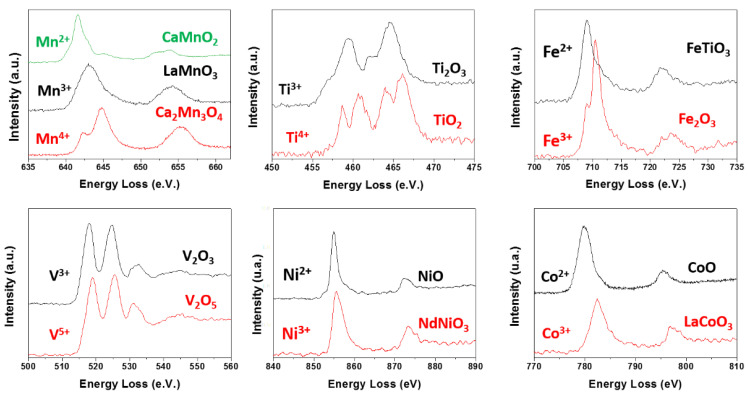
ML_2,3_ edges for several oxides containing M = Mn, Ti, Fe, V, Ni, and Co in different oxidation states.

Besides the structural and compositional information that can be obtained from TEM or STEM atomically resolved images, using aberration-corrected microscopy, it is also very important to get information on the oxidation states of the transition metal. In fact, electronic, magnetic, and catalytic properties as well as biological processes are controlled or tuned by the transition metal oxidation state, which can be obtained from the analysis of the absorption edges fine structure of transition metals and oxygen. Several methods have been described to correlate the oxidation states and the fine structure of the EELS M-L_2,3_ and O-K edges. A vast number of publications can be found in specialized journals [78,79,80]. Here, we will briefly focus on the L onset. The L_2,3_ edge arose from the excitation of 2p electrons into empty d levels. The position of the edge changes as a function of the oxidation state due to modification in the d electrons number [81]. To ascertain the edge position, it is very important to avoid any undesirable shift. For that purpose, simultaneous acquisition of the ZL and M-L_2,3_ edge using two energy windows is valuable. If the ZL peak is displaced from the 0.0 eV position, it can be moved to the correct value applying the same correction to the metal edge. Under this condition, we have studied different TMO systems (M = Mn, Ti, Fe, V, Ni, and Co) with M in various oxidation states (Figure 12). In each system, a shift of the L_2,3_ onset towards higher energy, when the oxidation state increased, was observed.

**Figure 13:**
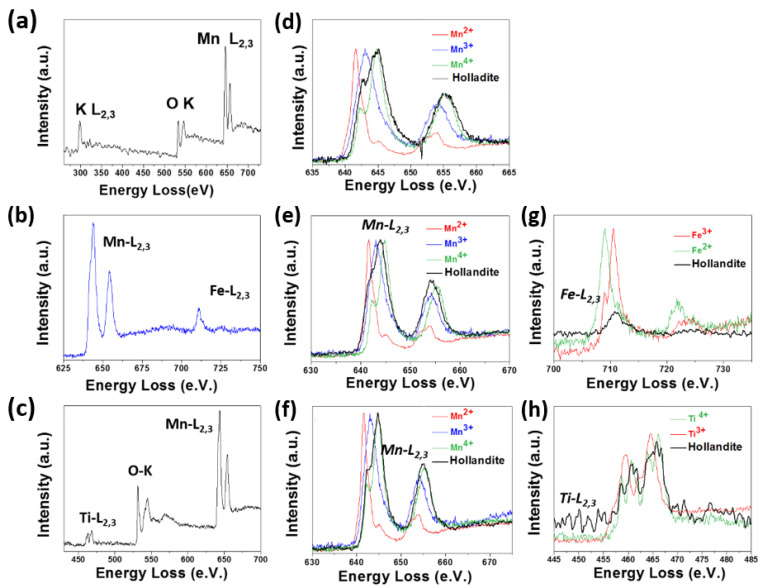
(**a**–**c**) EELS spectra characteristics of the undoped, and Fe-doped, and Ti-doped hollandites; (**d**) Mn L_2,3_ of undoped sample; (**e**) Mn L_2,3_ of Fe-doped sample; (**f)** Mn L_2,3_ of Ti-doped sample; (**g**) FeL_2,3_ of Fe-doped hollandite; (**h**) TiL_2,3_ of Ti-doped hollandite.

These spectra can be used as standards for the identification of the M oxidation state in TMO of interest. In this way, considering the standards for the Mn oxidation state, the coexistence of Mn^3+^ and Mn^4+^ has been verified in undoped (Figure 13a)and doped samples (Figure 13b,c) in the hollandite system, since the Mn edge appeared in an intermediate position between those corresponding to Mn^3+^ and Mn^4+^ (see Figure 13d–f) Figure 13b,d,g). On the other hand, Fe^3+^ (Figure 13g) and Ti^4+^ (Figure 13h) seemed to be present, since the edge position coincided with that of the standards for Fe^3+^ and Ti^4+^.

## 3. Conclusions

We have shown how TEM techniques can provide important morphological, dimensional, and microstructural information in several transition-metal-doped manganese hollandites, similar to that required in the several inorganic systems with biological applications referred to in the introduction section. Aberration-corrected electron microscopy allows access to atomically resolved TEM images. Even more, the combination of imaging and EELS in the STEM mode leads to the identification of minor dopants through the acquisition of atomically resolved chemical maps. In this way, how Fe and Ti substituted Mn in the hollandite structure has been shown. It has also been shown that ELNES analysis of the L_2,3_ edge, using different oxides as standards, can be very useful for studying the oxidation state of a certain transition metal. Clear differences in the position of the energy L_2,3_ onsets for the different Mn oxidation states, i.e., Mn^2+^, Mn^3+^, and Mn^4+^, as well as in its shape, can be observed. This analysis would provide precise information on the evolution of the Mn oxidation state in the MnO_2_ nano-oxides used in biological applications. In this context, the coexistence of Mn^4+^ and Mn^3+^ has been evidenced in hollandite oxides. The joint use of electron microscopy and spectroscopic techniques provides very useful structural and compositional information that, nowadays, can be obtained with unprecedented levels of resolution. Therefore, we have shown how combining imaging and spectroscopic capabilities at an atomic level reveals very precise structural and compositional details of TMO, giving answers to many of the turning points required for the understanding of the performance of TMO for biomedical uses.

## 4. Experimental

Average samples’ phase identification was assessed by X-ray diffraction (XRD) performed using a PANalytical X’pert PRO diffractometer operating with CuKα1 radiation in the Bragg−Brentano geometry at room temperature. Cation compositional analysis has been determined by means of electron microprobe microanalysis (EPMA), attached to a JEOL JXA-8900 microscope with around 20 areas of 1–5 μm. More local analysis has been performed by energy dispersive X-ray spectroscopy (EDS) in a JEOL JEM 2100 transmission electron microscope fitted with an EDS (OXFORD INCA) spectrometer. Atomically resolved transmission electron microscopy has been performed using an image spherical-aberration-corrected microscope JEOL JEM-GRAND ARM-300CF. Atomically resolved scanning transmission electron microscopy (STEM)-energy electron loss spectroscopy (EELS) characterization has been conducted in a probe spherical-aberration-corrected microscope JEOL JSM-ARM200F (Cold Emission Gun). According to preliminary studies in manganese perovskite-related oxides, the microscopes were operated between 60 and 120 kV, using low electron doses to minimize the damage of the samples. Inner and outer collection semiangles of 68 and 280 mrad were set for the acquisition of atomically resolved HAADF images. The JEOL JSM-ARM200F microscope was equipped with a GIF-QuantumERTM spectrometer, used for the EELS experiments (with a collection semiangle of 18 mrad and a convergence semiangle of 20.3 mrad). EELS chemical maps were acquired with a spatial resolution of ~0.08 nm over a total acquisition time of 1 min with an energy dispersion of 0.25 eV per channel. In order to study the oxidation states of the transition metals, electron energy loss near edge structure (ELNES) spectra were acquired using the spectrum line mode, with energy dispersions of 0.05 and 0.25 eV per channel and an acquisition time of 0.5 s over an average total number of 150 points and a pixel size of 1 Å. EELS principal component analysis was always performed on the EELS data set to de-noise the spectra by using the MSA plug-ins for Gatan DMS analysis toolbox [82]. The zero-loss peak was simultaneously acquired (10–5 s) using Dual EELS, meaning that the experimental signal was perfectly aligned and calibrated. The TEM image calculations were performed using the multislice method with the MacTempas software (M. A. O´Keefe, R. Kilaas. *Advances in High-Resolution Image Simulation.* Scanning Microscopy Suppl. 1988, 225–244.

## Data Availability

Not applicable.

## References

[1] Azab A.A., Ateia E.E., Esmail S.A. (2018). Comparative study on the physical properties of transition metal-doped (Co, Ni, Fe, and Mn) ZnO nanoparticles. Appl. Phys. A.

[2] Akbari A., Amini M., Tarassoli A., Eftekhari-Sis B., Ghasemian N., Jabbari E. (2018). Transition metal oxide nanoparticles as efficient catalysts in oxidation reactions. Nano Struct. Nano Objects.

[3] Sanchez C., Belleville P., Popall M., Nicole L. (2011). Applications of advanced hybrid organic–inorganic nanomaterials: From laboratory to market. Chem. Soc. Rev..

[4] Pałka K., Pokrowiecki R. (2018). Porous Titanium Implants: A Review. Adv. Eng. Mater..

[5] Yar A.Y., Aschauer U., Bowen P. (2018). Interaction of biologically relevant ions and organic molecules with titanium oxide (rutile) surfaces: A review on molecular dynamics studies. Colloids Surf. B Biointerfaces.

[6] Ke D., Vu A., Bandyopadhyay A., Bose S. (2018). Compositionally graded doped hydroxyapatite coating on titanium using laser and plasma spray deposition for bone implants. Acta Biomater..

[7] Trincă L.C., Mareci D., Souto R.M., Lozano-Gorrín A.D., Izquierdo J., Burtan L., Motrescu I., Vulpe V., Pavel G., Strungaru S. (2019). Osseointegration evaluation of ZrTi alloys with hydroxyapatite-zirconia-silver layer in pig’s tibiae. App. Surf. Sci..

[8] Kumari R., Majumdar J.D. (2018). Wear Behavior of Plasma Spray Deposited and Post Heat-Treated Hydroxyapatite (HA)-Based Composite Coating on Titanium Alloy (Ti-6Al-4V) Substrate. Met. Mater. Trans. A.

[9] Nosrati H., Mamoory R.S., Le D.Q.S., Bünger C.E. (2019). Preparation of reduced graphene oxide/hydroxyapatite nanocomposite and evaluation of graphene sheets/hydroxyapatite interface. Diam. Relat. Mater..

[10] Bose S., Vu A., Emshadi K., Bandyopadhyay A. (2018). Effects of polycaprolactone on alendronate drug release from Mg-doped hydroxyapatite coating on titanium. Mater. Sci. Eng. C.

[11] Beck T.J., Klust A., Batzill M., Diebold U., Di Valentin C., Tilocca A., Selloni A. (2005). Mixed dissociated/molecular monolayer of water on the TiO2(011)-(2×1) surface. Surf. Sci..

[12] Lindan P.J.D., Harrison N.M., Gillan M.J. (1998). Mixed Dissociative and Molecular Adsorption of Water on the Rutile (110) Surface. Phys. Rev. Lett..

[13] Schneider J., Ciacchi L.C. (2010). First principles and classical modeling of the oxidized titanium (0001) surface. Surf. Sci..

[14] Schneider J., Ciacchi L.C. (2010). A Classical Potential to Model the Adsorption of Biological Molecules on Oxidized Titanium Surfaces. J. Chem. Theory Comput..

[15] Kokubo T., Kim H.-M., Kawashita M. (2003). Novel bioactive materials with different mechanical properties. Biomaterials.

[16] Tas A.C. (2014). The use of physiological solutions or media in calcium phosphate synthesis and processing. Acta Biomater..

[17] Ferraris S., Yamaguchi S., Barbani N., Cristallini C., di Confiengo G.G., Barberi J., Cazzola M., Miola M., Vernè E., Spriano S. (2020). The mechanical and chemical stability of the interfaces in bioactive materials: The substrate-bioactive surface layer and hydroxyapatite-bioactive surface layer interfaces. Mater. Sci. Eng. C.

[18] Gossuin Y., Gillis P., Hocq A., Vuong Q.L., Roch A. (2009). Magnetic resonance relaxation properties of superparamagnetic particles. WIRE Nanomed. Nanobiotechnol..

[19] Nahrendorf M., Sosnovik D.E., Weissleder R. (2008). MR-optical imaging of cardiovascular molecular targets. Basic Res. Cardiol..

[20] Rümenapp C., Gleich B., Haase A. (2012). Magnetic Nanoparticles in Magnetic Resonance Imaging and Diagnostics. Pharm. Res..

[21] Xie J., Xu C., Xu Z., Hou Y., Young K.L., Wang S.X., Pourmand N., Sun S. (2006). Linking Hydrophilic Macromolecules to Monodisperse Magnetite (Fe3O4) Nanoparticles via Trichloro-s-triazine. Chem. Mater..

[22] McBain S.C., Yiu H.H.P., Dobson J. (2008). Magnetic nanoparticles for gene and drug delivery. Int. J. Nanomed..

[23] Vallet-Regí M., Ruiz-Hernández E. (2011). Bioceramics: From Bone Regeneration to Cancer Nanomedicine. Adv. Mater..

[24] Chomoucka J., Drbohlavova J., Huska D., Adam V., Kizek R., Hubalek J. (2010). Magnetic nanoparticles and targeted drug delivering. Pharmacol. Res..

[25] Drbohlavová J., Hrdý R., Adam V., Kizek R., Schneeweiss O., Hubalek J. (2009). Preparation and Properties of Various Magnetic Nanoparticles. Sensors.

[26] Tucek J., Zboril R., Petridis D. (2006). Maghemite Nanoparticles by View of Mössbauer Spectroscopy. J. Nanosci. Nanotechnol..

[27] Reshmi G., Kumar P.M., Malathi M. (2009). Preparation, characterization and dielectric studies on carbonyl iron/cellulose acetate hydrogen phthalate core/shell nanoparticles for drug delivery applications. Int. J. Pharm..

[28] Kumar A., Priyanka (2019). Environmentally benign pH-responsive cytidine-5′-monophosphate molecule-mediated akaganeite (5′-CMP-β-FeOOH) soft supramolecular hydrogels induced by the puckering of ribose sugar with efficient loading/release capabilities. New J. Chem..

[29] Chen M.-L., Shen L.-M., Chen S., Wang X.-W., Wang J.-H. (2013). In situ growth of β-FeOOH nanorods on graphene oxide with ultra-high relaxivity for in vivo magnetic resonance imaging and cancer therapy. J. Mater. Chem. B.

[30] Zeng L., Ren W., Zheng J., Wu A., Cui P. (2012). Synthesis of water-soluble FeOOH nanospindles and their performance for magnetic resonance imaging. Appl. Surf. Sci..

[31] Bae S., Lee S.W., Hirukawa A., Takemura Y., Jo Y.H. (2008). AC Magnetic-Field-Induced Heating and Physical Properties of Ferrite Nanoparticles for a Hyperthermia Agent in Medicine. IEEE Trans. Nanotechnol..

[32] Guisasola E., Asín L., Beola L., De La Fuente J.M., Baeza A., Vallet-Regí M. (2017). Beyond Traditional Hyperthermia: In Vivo Cancer Treatment with Magnetic-Responsive Mesoporous Silica Nanocarriers. ACS Appl. Mater. Interfaces.

[33] Darwish M.S.A., Kim H., Lee J.Y., Ryu C., Yoon J. (2019). Synthesis of Magnetic Ferrite Nanoparticles with High Hyperthermia Performance via a Controlled Co-Precipitation Method. Nanomaterials.

[34] Mazario E., Menendez N., Herrasti P., Cañete M., Connord V., Carrey J. (2013). Magnetic Hyperthermia Properties of Electrosynthesized Cobalt Ferrite Nanoparticles. J. Phys. Chem. C.

[35] Lee S.W., Bae S., Takemura Y., Shim I.-B., Kim T.M., Kim J., Lee H.J., Zurn S., Kim C.S. (2007). Self-heating characteristics of cobalt ferrite nanoparticles for hyperthermia application. J. Magn. Magn. Mater..

[36] Demirici Dönmez E., Manna P.K., Nickel R., Aktürk S., Van Lierop J. (2019). Comparative Heating Efficiency of Cobalt-, Manganese-, and Nickel-Ferrite Nanoparticles for a Hyperthermia Agent in Biomedicines. ACS Appl. Mater. Interfaces.

[37] Makridis A., Topouridou K., Tziomaki M., Sakellari D., Simeonidis K., Angelakeris M., Yavropoulou M.P., Yovos J.G., Kalogirou O. (2014). In vitro application of Mn-ferrite nanoparticles as novel magnetic hyperthermia agents. J. Mater. Chem. B.

[38] Dey C., Baishya K., Ghosh A., Goswami M.M., Ghosh A., Mandal K. (2017). Improvement of drug delivery by hyperthermia treatment using magnetic cubic cobalt ferrite nanoparticles. J. Magn. Magn. Mater..

[39] Du Y., Liu X., Liang Q., Liang X.-J., Tian J. (2019). Optimization and Design of Magnetic Ferrite Nanoparticles with Uniform Tumor Distribution for Highly Sensitive MRI/MPI Performance and Improved Magnetic Hyperthermia Therapy. Nano Lett..

[40] Umut E., Coşkun M., Pineider F., Berti D., Güngüneş H. (2019). Nickel ferrite nanoparticles for simultaneous use in magnetic resonance imaging and magnetic fluid hyper-thermia. J. Colloid Interface Sci..

[41] Huo J., Wei M. (2009). Characterization and magnetic properties of nanocrystalline nickel ferrite synthesized by hydrothermal method. Mater. Lett..

[42] Kang E., Park J., Hwang Y., Kang M., Park J.-G., Hyeon T. (2004). Direct Synthesis of Highly Crystalline and Monodisperse Manganese Ferrite Nanocrystals. J. Phys. Chem. B.

[43] Šepelák V., Bergmann I., Feldhoff A., Heitjans P., Krumeich F., Menzel D., Litterst F.J., Campbell S.J., Becker K.D. (2007). Nanocrystalline Nickel Ferrite, NiFe_2_O_4_:  Mechanosynthesis, Nonequilibrium Cation Distribution, Canted Spin Arrangement, and Magnetic Behavior. J. Phys. Chem. C.

[44] Virden A., Wells S., O’Grady K. (2007). Physical and magnetic properties of highly anisotropic cobalt ferrite particles. J. Magn. Magn. Mater..

[45] Colilla M., Manzano M., Izquierdo-Barba I., Vallet-Regí M., Boissiére C., Sanchez C. (2009). Advanced Drug Delivery Vectors with Tailored Surface Properties Made of Mesoporous Binary Oxides Submicronic Spheres. Chem. Mater..

[46] Abbasi A.Z., Prasad P., Cai P., He C., Foltz W.D., Amini M.A., Gordijo C.R., Rauth A.M., Wu X.Y. (2015). Manganese oxide and docetaxel co-loaded fluorescent polymer nanoparticles for dual modal imaging and chemotherapy of breast cancer. J. Control. Release.

[47] Haider M., Uhlemann S., Schwan E., Rose H., Kabius B., Urban K. (1998). Electron microscopy image enhanced. Nature.

[48] Krivanek O.L., Nellist P.D., Dellby N., Murfitt M.F., Szilagyi Z. (2003). Towards sub-0.5 A electron beams. Ultramicroscopy.

[49] Vallet-Regí M., Manzano M., González-Calbet J.M., Okunishi E. (2010). Evidence of drug confinement into silica mesoporous matrices by STEM spherical aberration corrected microscopy. Chem. Commun..

[50] Puig S., Ramos-Alonso L., Romero A.M., Martínez-Pastor M.T. (2017). The elemental role of iron in DNA synthesis and repair. Met..

[51] Wang H., Zhao S., Zhou J., Shen Y., Huang W., Zhang C., Rahaman M.N., Wang D. (2014). Evaluation of borate bioactive glass scaffolds as a controlled delivery system for copper ions in stimulating osteogenesis and angiogenesis in bone healing. J. Mater. Chem. B.

[52] Hambidge M. (2000). Human Zinc Deficiency. J. Nutr..

[53] Schroeder H.A., Balassa J.J., Tipton I.H. (1970). Essential trace metals in man: Molybdenum. J. Chronic Dis..

[54] Crans D., Trujillo A.M., Pharazyn P.S., Cohen M.D. (2011). How environment affects drug activity: Localization, compartmentalization and reactions of a vanadium insulin-enhancing compound, dipicolinatooxovanadium(V). Coord. Chem. Rev..

[55] Brokesh A.M., Gaharwar A.K. (2020). Inorganic Biomaterials for Regenerative Medicine. ACS Appl. Mater. Interfaces.

[56] Garino N., Sanvitale P., Dumontel B., Laurenti M., Colilla M., Izquierdo-Barba I., Cauda V., Vallet-Regì M. (2019). Zinc oxide nanocrystals as a nanoantibiotic and osteoinductive agent. RSC Adv..

[57] Natalio F., Andre R., Hartog A.F., Stoll B., Jochum K.P., Wever R., Tremel W. (2012). Vanadium pentoxide nanoparticles mimic vanadium haloperoxidases and thwart biofilm formation. Nat. Nanotechnol..

[58] Qi Y., Qi H., He Y., Lin W., Yongli Q., Qin L., Hu Y., Chen L., Liu Q., Sun H. (2017). Strategy of Metal–Polymer Composite Stent to Accelerate Biodegradation of Iron-Based Biomaterials. ACS Appl. Mater. Interfaces.

[59] Shi M., Chen Z., Farnaghi S., Friis T., Mao X., Xiao Y., Wu C. (2016). Copper-doped mesoporous silica nanospheres, a promising immunomodulatory agent for inducing osteogenesis. Acta Biomater..

[60] Prohaska J.R. (1987). Functions of trace elements in brain metabolism. Physiol. Rev..

[61] Bae Y.-J., Kim M.-H. (2008). Manganese Supplementation Improves Mineral Density of the Spine and Femur and Serum Oste-ocalcin in Rats. Biol. Trace Elem. Res..

[62] Bose S., Fielding G., Tarafder S., Bandyopadhyay A. (2013). Understanding of dopant-induced osteogenesis and angiogenesis in calcium phosphate ceramics. Trends Biotechnol..

[63] Pabbruwe M.B., Standard O.C., Sorrell C.C., Howlett C.R. (2004). Bone formation within alumina tubes: Effect of calcium, manganese, and chromium dopants. Biomaterials.

[64] Lu J., Ma S., Sun J., Xia C., Liu C., Wang Z., Zhao X., Gao F., Gong Q., Song B. (2009). Manganese ferrite nanoparticle micellar nanocomposites as MRI contrast agent for liver imaging. Biomaterials.

[65] Bae K.H., Lee K., Kim C., Park T.G. (2011). Surface functionalized hollow manganese oxide nanoparticles for cancer targeted siRNA delivery and mag-netic resonance imaging. Biomaterials.

[66] Prasad P., Gordijo C.R., Abbasi A.Z., Maeda A., Ip A., Rauth A.M., DaCosta R.S. (2014). Wu, X.Y. Multifunctional Albumin–MnO_2_ Nanoparticles Modulate Solid Tumor Microenvironment by Attenuating Hypoxia, Acidosis, Vascular Endothelial Growth Factor and Enhance Radiation Response. ACS Nano.

[67] Luo X.-L., Xu J.-J., Zhao W., Chen H.-Y. (2004). A novel glucose ENFET based on the special reactivity of MnO_2_ nanoparticles. Biosens. Bioelectron..

[68] Fan W., Bu W., Shen B., He Q., Cui Z., Liu Y., Zheng X., Zhao K., Shi J. (2015). Intelligent MnO_2_ Nanosheets Anchored with Upconversion Nanoprobes for Concurrent pH-/H_2_O_2_-Responsive UCL Imaging and Oxygen-Elevated Synergetic Therapy. Adv. Mater..

[69] Chen Q., Feng L., Liu J., Zhu W., Dong Z., Wu J., Liu Z. (2016). Intelligent Albumin–MnO_2_ Nanoparticles as pH-/H_2_O_2_-Responsive Dissociable Nanocarriers to Modulate Tumor Hypoxia for Effective Combination Therapy. Adv. Mater..

[70] Yang G., Xu L., Chao Y., Xu J., Sun X., Wu Y., Peng R., Liu Z. (2017). Hollow MnO_2_ as a tumor-microenvironment-responsive biodegradable nano-platform for combination therapy favoring antitumor immune responses. Nat. Commun..

[71] Wu M., Hou P., Dong L., Cai L., Chen Z., Zhao M., Li J. (2019). Manganese dioxide nanosheets: From preparation to biomedical applications. Int. J. Nanomed..

[72] Chen Y., Cong H., Shen Y., Yu B. (2020). Biomedical application of manganese dioxide nanomaterials. Nanotechnology.

[73] Azor-Lafarga A., Ruiz-González L., Parras M., Portehault D., Sanchez C., González-Calbet J.M. (2018). Modified Synthesis Strategies for the Stabilization of low n TinO2n–1 Magnéli Phases. Chem. Record.

[74] Portehault D., Maneeratana V., Candolfi C., Oeschler N., Veremchuk I., Grin Y., Sanchez C., Antonietti M. (2011). Facile General Route toward Tunable Magnéli Nanostructures and Their Use AS Thermoelectric Metal Oxide/Carbon Nanocomposites. ACS Nano.

[75] Garcia M.A., Merino J.M., Pinel E.F., Quesada A., de la Venta J., Gonzalez M.L.R., Castro G., Crespo P., Llopis J., Gonzalez-Calbet J.M. (2007). Magnetic Properties of ZnO Nanoparticles. Nano Lett..

[76] Gómez-Recio I., Azor-Lafarga A., Ruiz-González M.L., Hernando M., Parras M., Calvino J.J., Fernández-Díaz M.T., Portehault D., Sanchez C., González-Calbet J.M. (2020). Unambiguous localization of titanium and iron cations in doped manganese hollandite nanowires. Chem. Commun..

[77] Egerton R.F. (2008). Electron energy-loss spectroscopy in the TEM. Rep. Prog. Phys..

[78] Colliex C., Manoubi T., Ortiz C. (1991). Electron-energy-loss-spectroscopy near-edge fine structures in the iron-oxygen system. Phys. Rev. B.

[79] Garvie L.A.J., Craven A.J. (1994). High-resolution parallel electron energy-loss spectroscopy of Mn L2,3-edges in inorganic manganese compounds. Phys. Chem. Miner..

[80] Varela M., Oxley M., Luo W., Tao J., Watanabe M., Lupini A., Pantelides S.T., Pennycook S.J. (2009). Atomic-resolution imaging of oxidation states in manganites. Phys. Rev. B.

[81] Krivanek O., Disko M., Taftø J., Spence J. (1982). Electron energy loss spectroscopy as a probe of the local atomic environment. Ultramicroscopy.

[82] Watanabe M., Okunishi E., Ishizuka K. (2009). Analysis of spectrum-imaging datasets in atomic-resolution electron microscopy. Microsc. Anal..

